# The gut microbiome and cancer response to immune checkpoint inhibitors

**DOI:** 10.1172/JCI184321

**Published:** 2025-02-03

**Authors:** Francesca S. Gazzaniga, Dennis L. Kasper

**Affiliations:** 1 Department of Pathology and Krantz Family Center for Cancer Research, Massachusetts General Hospital and Harvard Medical School, Boston, Massachusetts, USA.; 2 Department of Immunology, Blavatnik Institute, Harvard Medical School, Boston, Massachusetts, USA.

## Abstract

Immune checkpoint inhibitors (ICIs) are widely used for cancer immunotherapy, yet only a fraction of patients respond. Remarkably, gut bacteria impact the efficacy of ICIs in fighting tumors outside of the gut. Certain strains of commensal gut bacteria promote antitumor responses to ICIs in a variety of preclinical mouse tumor models. Patients with cancer who respond to ICIs have a different microbiome compared with that of patients who don’t respond. Fecal microbiota transplants (FMTs) from patients into mice phenocopy the patient tumor responses: FMTs from responders promote response to ICIs, whereas FMTs from nonresponders do not promote a response. In patients, FMTs from patients who have had a complete response to ICIs can overcome resistance in patients who progress on treatment. However, the responses to FMTs are variable. Though emerging studies indicate that gut bacteria can promote antitumor immunity in the absence of ICIs, this Review will focus on studies that demonstrate relationships between the gut microbiome and response to ICIs. We will explore studies investigating which bacteria promote response to ICIs in preclinical models, which bacteria are associated with response in patients with cancer receiving ICIs, the mechanisms by which gut bacteria promote antitumor immunity, and how microbiome-based therapies can be translated to the clinic.

## Introduction

The field of the microbiome and immunotherapy has skyrocketed since 2015 when two studies demonstrated a fascinating phenomenon in mice: certain gut bacteria could enhance the efficacy of immune checkpoint inhibitor (ICI) therapy in tumors outside of the gut (1, 2). ICIs are a type of cancer immunotherapy in which antibodies that block ICI molecules reinvigorate immune cells to mount a robust anticancer attack. The current approved ICIs consist of blocking antibodies against programmed cell death protein 1 (PD-1), programmed cell death ligand 1 (PD-L1), cytotoxic T-lymphocyte associated protein 4 (CTLA-4), and lymphocyte activation gene 3 (LAG3), and these checkpoint inhibitors are approved for over twenty different cancer types (3). Though ICIs are the frontline treatment for multiple cancers, including advanced cutaneous melanoma, non–small cell lung cancer (NSCLC), and renal cell carcinoma (RCC) among others, the durable progression-free survival in these and other tumors remains less than 50%, highlighting the critical need to understand what affects efficacy and what can be done to improve it. Using mouse sarcoma models to investigate the mechanisms of action for ICIs, one group found that antibiotic treatment abrogated the antitumor effects of anti–CTLA-4 therapy. Independently, using mouse melanoma models, another group found that microbiomes from different animal vendors affected the efficacy of anti–PD-L1 therapy. Both studies identified specific bacterial species that could enhance the efficacy of ICIs. *Bacteroides fragilis* or *Bacteroides thetaiotaomicron* could enhance the efficacy of anti–CTLA-4 in the mouse model of sarcoma (2), and *Bifidobacterium* species could enhance the efficacy of anti–PD-L1 in a mouse model of melanoma (1), Therefore, when these two studies, which used different tumor models and were investigating different observations (antibiotic treatment vs. different vendor microbiomes), independently arrived at the same conclusion that the composition of the gut microbiome affects efficacy of ICIs, the field of the microbiome and cancer immunotherapy expanded markedly.

## Bacteria that promote response to ICIs in mice

Multiple preclinical studies have demonstrated a cause-and-effect relationship between gut bacteria and response to ICIs. Some studies use a defined consortium of bacteria to promote antitumor immunity to PD-1/PD-L1 blockade in preclinical models. These antitumor consortia include a mix of *Bifidobacterium* (1), a mix of *Clostridiales* (4), and an 11-strain mix containing *Parabacteroides*, *Alistipes*, *Paraprevotella*, *Bacteroides*, *Eubacterium*, *Clostridiales*, *Phascolarctobacterium*, and *Fusobacterium* (5). Other studies have identified individual strains of bacteria sufficient to promote antitumor immunity to checkpoint inhibitors. *Bifidobacterium breve* (1), *Bifidobacterium longum* (1), *Akkermansia muciniphila* (6), *Alistipes indistinctus* (6), *Enterococcus hirae* (6, 7), *Enterococcus faecium* (7), *Enterococcus durans* (7), *Enterococcus mundtii* (7), *Coprobacillus cateniformis* (8), *Erysipelatoclostridium ramosum* (8), *Lactobacillus gallinarium* (9), *Lactobacillus rhamnosus GG* (10), *Roseburia intestinalis* (11), and *Faecalibacterium prausnitzii* (12) have all been shown to promote antitumor responses to PD-1/PD-L1 blockade in mice. Additionally, *B*. *fragilis* (2), *B*. *thetaiotaomicron* (2), *Burkholderia cepacian* (2), *Bifidobacterium pseudolongum* (13), *Lactobacillus johnsonii* (13), *Olsenella sp*. (13) and *E*. *faecium* (7) promote antitumor responses to anti–CLTA-4 treatment in mice. Table 1 summarizes bacterial species that promote antitumor immunity to ICIs in different preclinical mouse tumor models. The antitumor effects of these bacterial species are demonstrated in germ-free (GF) mice that have been monocolonized with bacteria (2, 8, 13), in antibiotic pretreated mice that subsequently receive oral gavage of the bacteria (2, 6–8), or in mice receiving oral gavage on top of their conventional mouse microbiota (1, 7, 9, 11). On the other hand, response to ICIs is abrogated in mice originating from Taconic Biosciences versus The Jackson Laboratory (1), GF mice (2, 8, 13), mice treated with antibiotics (2, 6, 8), or mice receiving the probiotics *Bifidobacterium longum 35624* or *Lactobacillus rhamnosus GG* (14). Notably *L*. *rhamnosus GG* has been shown to have both pro- and antitumor effects in response to ICI in mice. These differences could be due to the different tumor lines used, different treatments (anti–PD-1 vs. anti–PD-L1), different sources of the bacteria, or the different microbiomes of mice housed in different facilities. Understanding the mechanisms by which specific bacteria impact antitumor immunity may increase the reproducibility of their effects in different contexts. These studies clearly demonstrate that the composition of the gut microbiota can impact the antitumor response to ICIs in mice. These preclinical models can be used to investigate the mechanistic relationship between certain gut microbes and the immune response to ICIs.

## Bacteria associated with cancer immunotherapy responses in patients

Importantly, the relationship between the gut microbiome and response to immunotherapy can also be found through analysis of patient samples. Certain members of the gut microbiome are associated with response to ICIs in melanoma (15–22), NSCLC (6, 23–28), RCC (6, 23, 29), hepatocellular carcinoma (30–34), thoracic carcinoma (35), and urothelial (6), gastrointestinal (36), and hepatobiliary cancers (37), which has been recently reported in ref. 38. Notably, these studies, which investigate various cancers and treatments, identified different bacteria associated with response. Focusing in on the first three studies describing associations between the composition of the microbiota and response to anti–PD-1 therapy, one study identified *Akkermansia muciniphila*, *Alistipes spp*, *Ruminococcus spp*, and *Eubacterium spp* as enriched in responders with NSCLC (6). In patients with melanoma, one study identified *Faecalibacterium* to be enriched in responders (17), and another identified *B*. *longum*, *Collinsella aerofaciens*, and *E*. *faceium* to be enriched in responders (18). Though different bacteria were associated with response in each of these studies, these studies demonstrated that fecal microbiota transplants (FMTs) from patients into GF (6, 17, 18) or antibiotic-treated (6) mice can transfer the patient tumor response to ICIs. Mice colonized with responder melanoma stool and subsequently implanted with melanoma tumors responded to checkpoint therapy (17, 18). Similarly, mice colonized with responder RCC stool and subsequently implanted with an RCC cell line responded to anti–CTLA-4 therapy (6). Interestingly, the patient tumor and mouse tumor did not have to match to transfer the patient response to ICI to the mice. Mice colonized with responder NSCLC stool and subsequently colonized with a sarcoma line also responded to anti–CTLA-4 therapy (6), and we have observed that mice colonized with responder melanoma stool and subsequently implanted with a colon carcinoma also respond to anti–PD-L1 therapy (8). Conversely, mice receiving FMTs from patients who did not respond to anti–PD-1 therapy did not respond to checkpoint inhibitors (6, 17, 18). Though not all patient tumor responses could be transferred to mice via FMT (18), these studies show that for some patients, the fecal microbiota strongly affects the response to immunotherapy.

The differences in bacterial species associated with response could be due to several factors. Gut bacteria have been shown to have many different immunomodulatory effects (39), and different bacterial species could impact antitumor immunity by different mechanisms, some of which are described below. Additionally multiple species or genera could affect the same immune mechanism (39). Therefore, instead of needing one specific species to promote antitumor immunity, having one of several different species may be sufficient to promote a response. Along these lines, the methods for measuring microbial composition could impact the genera or species identified. 16S rRNA sequencing is cost effective, relatively quick, and straightforward to analyze. However, it does not give species-level resolution for many species. Instead, metagenomic analysis of bacterial DNA from stool is being used to obtain species-level resolution and to identify bacterial genes, as opposed to species, that associate with response (40). Metagenomic analysis, therefore, might identify genes associated with response that could be shared by multiple species. Notably, diet impacts the composition of the gut microbiota (41). Therefore, the differences in species associated with response in different studies could be affected by diets common in that region. Regardless, while the specific bacterial species may vary in different populations, it has been found worldwide that the composition of the gut microbiome is associated with response to immunotherapy in many cancers.

## Antibiotics affect antitumor immunity in mice and patients

Several studies have shown that antibiotic cocktails or individual antibiotics abrogate the antitumor effects of ICIs in preclinical models. A cocktail of ampicillin, colistin, and streptomycin (ACS) abrogates response to anti–PD-1 therapy in MCA205 tumors (6) and anti–CTLA-4 therapy for MCA205, RET, and MC38 tumors (2). A cocktail of ampicillin, metronidazole, vancomycin, and neomycin abrogates the efficacy of PD-1/PD-L1 blockade in MC38 tumors (8). As single agents, colistin reduces the antitumor effects of anti–CTLA-4 treatment on MCA205 tumors (2), and ampicillin, metronidazole, and vancomycin reduce antitumor responses to anti–PD-L1 treatment in MC38 tumors (8). Similarly antibiotic usage in patients has been associated with worse survival in RCC (6), NSCLC (6, 42), and triple-negative breast cancer (43). Interestingly, reducing antitumor immunity promoting bacteria may not be the only mechanism by which antibiotics reduce the effect of ICIs. Bacteria that grow in the presence of antibiotics or soon after antibiotics have stopped could have negative effects on antitumor immunity. ACS treatment induces gut dysbiosis, reduces MADCAM-1 expression in the ileum, and increases tumor infiltrating regulatory T17 cells. Colonization with *E*. *clostridioformis*, a bacterium that increases in abundance with ACS treatment, drives α4β7 CD4 regulatory T17 cells into the tumor. This increase in T17 regulatory cells in the tumors either by anti-MadCam1 or anti-α4β7 blocks response to anti–PD-1 treatment in MCA205 and 4T1 mouse tumor models (44). Therefore, antibiotic treatments may inhibit antitumor immunity by reducing bacteria that promote antitumor immunity and by enabling the growth of bacteria that inhibit antitumor immunity.

## FMTs in mice and patients with cancer

Because the composition of the gut microbiome is different in patients with cancer who respond to ICIs and FMTs from responder patients into mice promoted antitumor responses (6, 8, 17, 18), FMTs are being explored as a potential cancer therapy. Initially two clinical trials demonstrated that FMTs from patients with melanoma who responded to anti–PD-1 therapy could overcome resistance in about a third of patients who had progressed on treatment (45, 46). These proof-of-concept studies clearly show that the gut microbiota can promote response to ICIs in some patients. However, while these small, 10- to 15-patient study sizes, demonstrate the potential for FMTs, they were not designed to determine if the unpredictable efficacy was due to variations between fecal donors or inherent differences between the recipients. More recent studies have focused on increasing the efficacy of FMTs. The response to FMT + anti–PD-1 is increased to 65% when patients with advanced melanoma receive an FMT prior to their first dose of anti–PD-1 (47). Recently, a metagenomic analysis of fecal samples was performed on 872 patients with NSCLC and genitourinary, and colorectal cancer to develop a qPCR-based test of 21 bacterial strains that can stratify patients with NSCLC between those that have a good prognosis for survival with immunotherapy and those that might benefit from additional therapies (24). This scoring could be useful in the future to screen potential samples for those more likely to promote a response to immunotherapy. Though many clinical trials are underway (38), FMTs may only be a stopgap until more reliable therapies are developed (48). Fecal transplants contain billions of live organisms, making them difficult to regulate or standardize. Furthermore, though fecal transplants are relatively safe and are very effective for treating *Clostridiodes difficile* infections, there is a risk of bacterial infections, including sepsis (49). For patients such as those with melanoma, who only receive ICIs, the potential benefits may outweigh the risks. However, treatments for many other cancers combine ICIs with chemotherapies that could dampen the immune response and might increase the risks from fecal transplants. Therefore, understanding the specific bacteria that promote antitumor immunity, and their mechanisms of action, could lead to more reliable and safe therapies.

## Effect of probiotics on outcomes of patients with cancer

Though many studies have demonstrated that oral gavage of individual strains of live bacteria can increase the efficacy of ICIs in mice (1, 2, 7–9, 11–13), probiotics have had variable effects in the clinic. Two studies found benefits with the probiotic, *Clostridium butyricum* in patients with RCC receiving nivolumab plus ipilimumab (50) and in patients with RCC receiving cabozantinib and nivolumab (51). On the other hand, another study found worse survival was associated with taking off-the-shelf probiotics in melanoma, and supplementing mice with a commercially available Bifidobacterium-based probiotic increased tumor sizes (14). Furthermore, a small, 14-patient study suggested that preconditioning with antibiotics prior to taking a Firmicutes-enriched probiotic also showed worse survival in melanoma (52). As clinical trials for several strains or cocktails of strains that promoted antitumor immunity in mice are underway (38), understanding how antibiotic treatment or other factors impact the efficacy of these strains is essential to maximize efficacy in patients. Gut bacteria are incredibly sensitive to their environment, with diet, medications, and exercise all affecting the composition of the gut microbiota (41, 53). Therefore, a probiotic strain may produce metabolites that promote antitumor immunity in a controlled lab setting but may have variable effects in patients with different lifestyles and cancer treatments.

## Bacterial metabolites as cancer therapies

**Figure 1:**
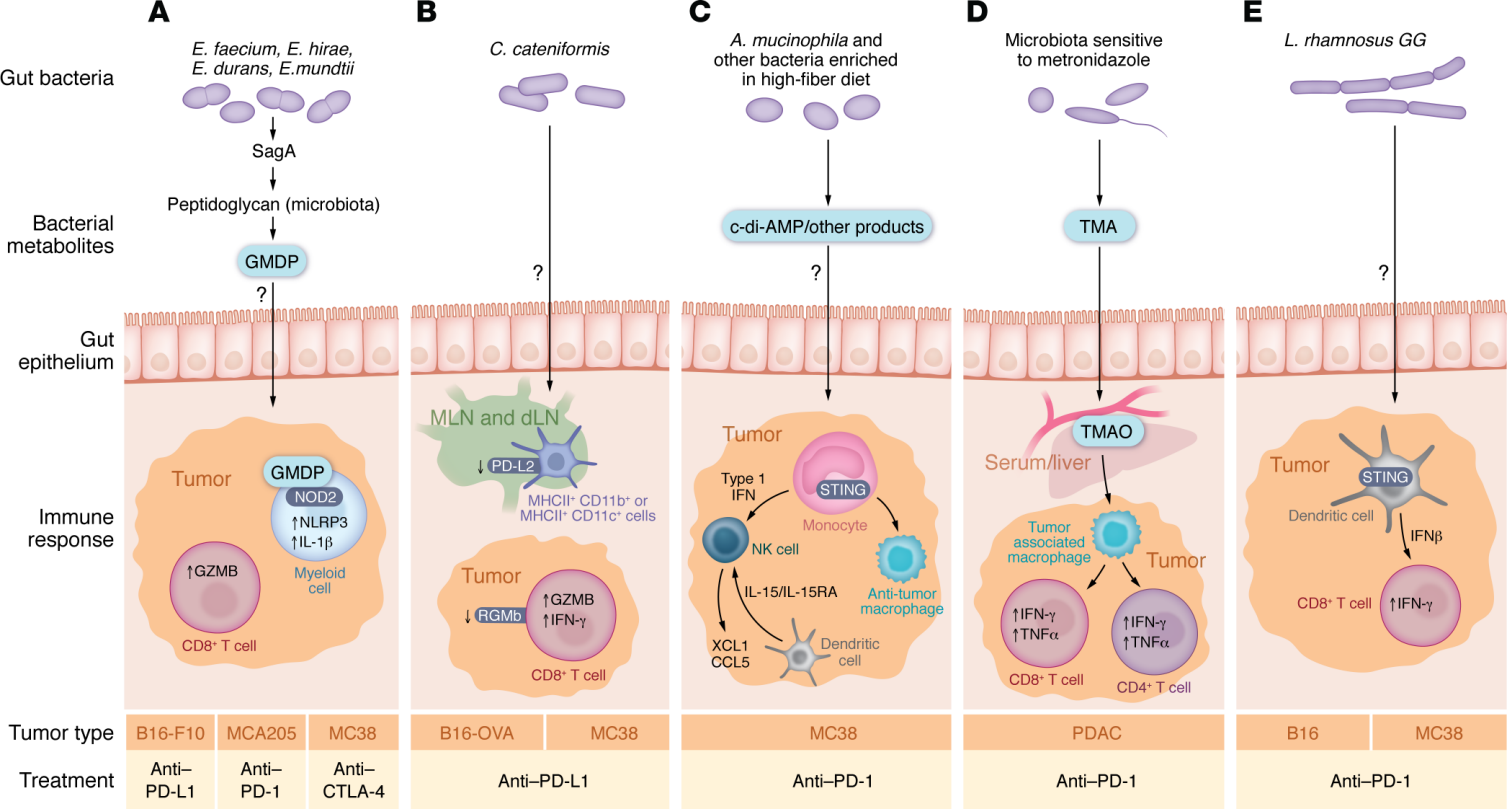
Mechanisms of gut bacteria–mediated antitumor immunity. (**A**) *E*. *faecium*, *E*. *hirae*, *E*. *durans*, and *E*. *mundtii* release orthogolgs of SagA, a peptidoglycan hydrolase that breaks muramyl bonds in peptidoglycan of other gut bacteria to release GMDP. GMDP signals through NOD2 on myeloid cells to increase transcription of IL-1b and NLRP3 and increase granzyme B^+^ (GZMB^+^) CD8^+^ T cells in the tumor (7). Whether GMDP released by gut bacteria travel from the tumor or immune cells from the gut that have been exposed to GMDP travel to the tumors is unknown. (**B**) *C*. *cateniformis* contains a surface metabolite that suppresses PD-L2 expression on MHCII^+^CD11b^+^ and MHCII^+^CD11c^+^ immune cells in the mesenteric and tumor-draining lymph nodes (MLNs and dLNs). Blockade of PD-L2/RGMb interactions increases tumor-infiltrating GZMB^+^ and IFN-γ^+^CD8^+^ T cells in the tumors to promote antitumor immunity to anti–PD-L1 (8). How *C*. *cateniformis* suppresses PD-L2, whether the microbial surface metabolite or cells that interact with *C*. *cateniformis* travel to the dLN, and how the gut microbiome impacts RGMb expression are unknown. (**C**) *A*. *mucinophila* and other bacteria that increase in abundance on a high-fiber diet release c-di-AMP and other products. These products signal through cGAS/STING in monocytes, stimulating antitumor macrophages and releasing type 1 IFNs that stimulate NK cells to release XCL1 and CCL5 and increase tumor-infiltrating dendritic cells to release IL-15 and its receptor IL-15RA. This monocyte-NK-DC crosstalk promotes antitumor responses to anti–PD-1 (55). Whether microbially derived STING agonists or monocytes that have interacted with microbially derived STING agonists in the gut travel to the tumors is unknown. (**D**) Gut bacteria sensitive to oral metronidazole release TMA, which gets converted into TMAO in the liver, enters the blood stream, and stimulates tumor-associated macrophages to increase IFN-γ^+^ TNF-α^+^ CD8^+^ and CD4^+^ T cells in the tumor in a type 1 IFN-dependent manner; this increases response of pancreatic ductal adenocarcinoma (PDAC) tumors to anti–PD-1 therapy (40). (**E**) *L*. *rhamnosus GG* was also shown to signal through cGAS/STING on dendritic cells to release IFN-β and increase IFN-γ^+^CD8^+^ T cells in tumors, promoting antitumor immunity to anti–PD-1 treatment (10). The identity of the microbial metabolite from *L*. *rhamnosus GG*, and whether the metabolite or cells that interacted with *L*. *rhamnosus GG* travel from the gut to the tumor, are unknown. Furthermore, it is unclear why *L*. *rhamnosus GG* promotes antitumor immunity in some conditions, but not others (10, 14).

**Figure 2:**
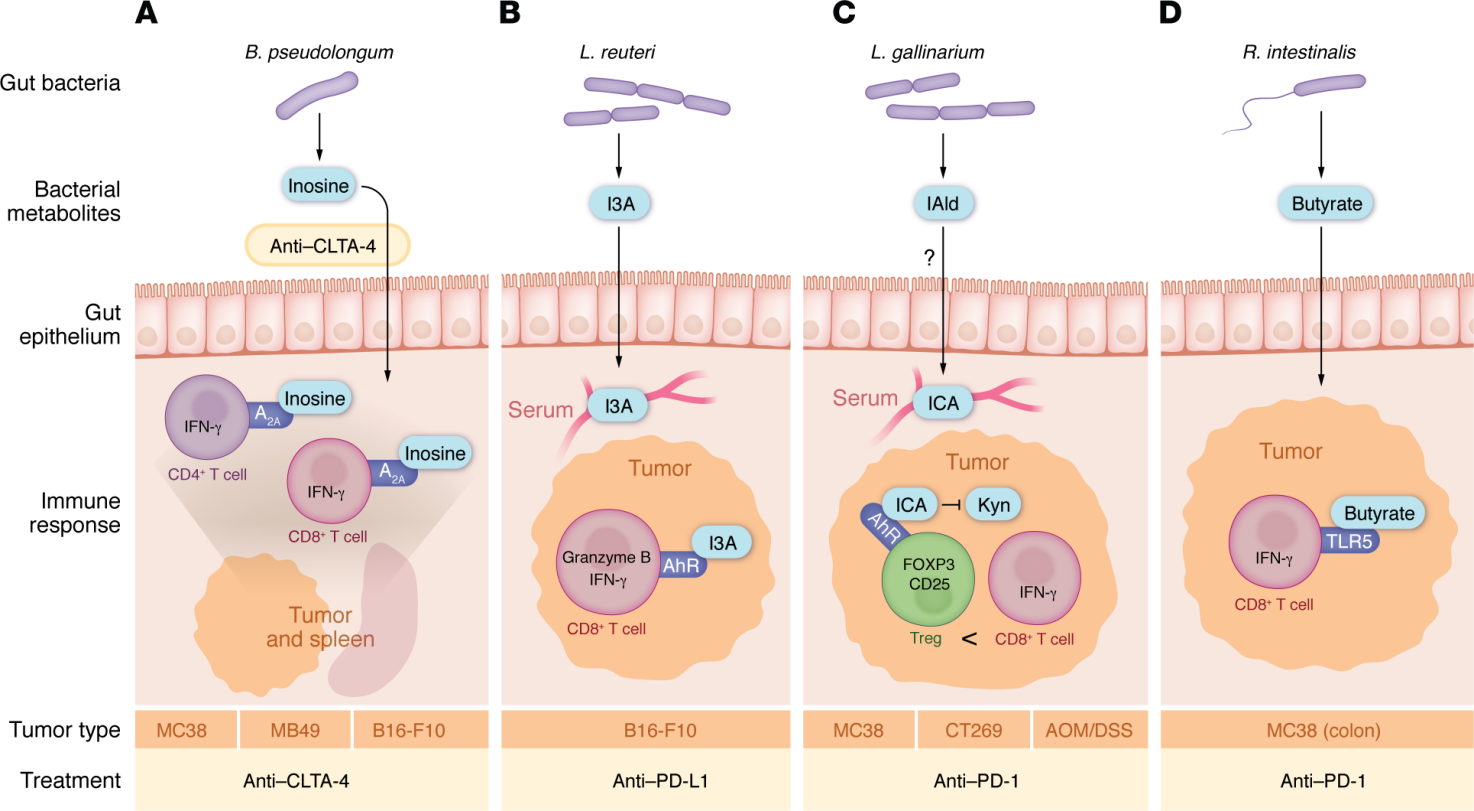
Gut bacterial metabolites that directly impact T cells in tumors. (**A**) *B*. *pseudolongum* releases inosine. Upon treatment with anti–CTLA-4, inosine enters the bloodstream and signals through the adenosine receptor (A_2A_) to increase IFN-γ^+^CD4^+^ and CD8^+^ T cells to promote response to anti–CTLA-4 (13). (**B**) *L*. *reuteri* releases indole-3-aldehyde (I3A), which enters the bloodstream and signals through the aryl hydrocarbon receptor (AhR) to promote tumor-infiltrating GZMB^+^ and IFN-γ^+^CD8^+^ T cells and increase response to anti–PD-L1 treatment (54). *L*. *reuteri* also appears to translocate to the tumor to promote antitumor immunity, though how it translocates to the tumor without inducing an infection response is unknown (54). (**C**) *L*. *gallinarium* produces indole-3-carboxaldehyde, which gets converted in the serum to indole-3-carboxylic acid (ICA), which blocks kynurenine (Kyn) signaling through the AhR receptor. This decreases the amount of tumor-infiltrating Tregs, resulting in more IFN-γ^+^CD8^+^ T cells in the tumors; this in turn promotes antitumor responses to anti–PD-1 therapy in tumors implanted subcutaneously and in tumors arising in the gut by AOM/DSS-induced colitis (9). (**D**) In tumors in the colon, *R*. *intestinalis* releases butyrate that signals through TLR5 to induce IFN-γ+CD8^+^ T and increases response to anti–PD-1 treatment. Whether this mechanism works in tumors outside of the gut remains unclear (11).

To circumvent the inherent variability in live bacterial treatments, bacterial metabolites have been explored as cancer immunotherapies. Figure 1 depicts the mechanisms by which specific gut bacteria promote antitumor immunity via antigen-presenting cells, and Figure 2 depicts direct T cell mechanisms for bacterially mediated antitumor immunity.

In preclinical mouse models, several studies have identified secreted bacterial metabolites that promote antitumor immunity. *E*. *faceium*, *E*. *hirae*, *E*. *durans*, and *E*. *mundtii* release orthologs of peptidoglycan hydrolase that breaks peptidoglycan bonds to generate muropeptides such as GlcNac-muramyl dipeptide (GMDP). GMDP signals through NOD2 on myeloid cells to release IL-1b and NLRP3 to induce cytotoxic granzyme B^+^CD8^+^ T cells in the tumor and enhances the efficacy of anti–CTLA-4, anti–PD-1, and anti–PD-L1 in different mouse tumor models (7). *B*. *pseudolongum*, on the other hand, promotes antitumor immunity through inosine production. In *B*. *pseudolongum*–colonized mice, treatment with anti–CTLA-4 enables inosine to enter the bloodstream and signal through the adenosine A_2A_ receptor on T cells in the spleen and tumors to release IFN-γ and promote antitumor immunity in both xenograft and genetic mouse models (13).

Two different *Lactobacillus* species release tryptophan metabolites to promote antitumor immunity. *Lactobacillus reuteri* in the gut and tumor releases indole-3-aldehyde (I3A), which signals through aryl hydrocarbon receptor (AhR) on CD8^+^ T cells to release IFN-γ and promote antitumor immunity to anti–PD-1 therapy (54). *Lactobacillus gallinarium* releases indole-3-carboxylic acid (ICA), which competes with kynurenine to bind AhR and inhibit tumor-infiltrating Tregs, resulting in increased IFN-γ^+^ CD8^+^ T cells in tumors and increased response to anti–PD-1 treatment (9).

Gut microbiota from a high-fiber diet, including *Akkermansia muciniphila*, release ci-di-AMP and other factors that signal through cGAS/STING to remodel macrophages and NK-dendritic cell interactions to promote antitumor immunity to PD-1/PD-L1 blockade (55). Similarly, *Lactobacillus rhamnosus GG* signals via cGAS/STING to induce dendritic cells to release IFN-β to increase IFN-γ CD8^+^ T cells and reduce tumor sizes in anti–PD-1–treated mice (10). *Roseburia intestinalis* releases butyrate to increase cytotoxic CD8^+^ T cells to produce IFN-γ and granzyme B and increase response of colonic tumors to anti–PD-1 (11). Gut bacteria sensitive to metronidazole metabolize choline into trimethylamine (TMA), which gets converted into TMA N-oxide (TMAO), which in turn stimulates tumor-associated macrophages to promote IFN-γ^+^ T cells in a type 1 interferon manner to promote antitumor immunity to ICI in a pancreatic cancer model (40).

In addition to secreted metabolites, surface metabolites on bacteria have immunomodulatory effects that could be harnessed for immunotherapy. Recently, we showed that a surface extract from *C*. *cateniformis* can suppress PD-L2 in vitro and increases the efficacy of PD-1/PD-L1 blockade in preclinical models (8).

Beyond administering the bacterial metabolite as a potential therapy, there are other approaches to increase the efficacy of microbiome-based therapies by administering the bacterial metabolite as a therapy or administering molecules that target the mechanism of action of the bacterial metabolite. The bacterial metabolites inosine (13), I3A (54), ICA (9), TMA (40), TMAO (40), and butyrate (11) have been shown to increase the efficacy of ICIs in preclinical models. Targeting the downstream effects of microbial metabolites has also been explored to increase response to ICI. Administration of MDP, a NOD2 agonist, increases antitumor immunity to PD-1/PD-L1 blockade (7), and using blocking antibodies against PD-L2/RGMb overcomes microbiome-mediated resistance in multiple mouse tumor models (8). Importantly, anti–PD-L2 treatment overcame resistance to monotherapy in mice colonized with stool samples from patients who did not respond. Because several studies have shown that colonization with nonresponder stool samples reduces antitumor immunity to ICIs, testing new therapies in the context of nonresponder microbiomes could be a useful strategy to identify novel therapies that overcome microbiome-dependent resistance to treatment. As more microbe-mediated mechanisms of antitumor immunity are discovered, using antibodies or drugs that target these pathways have the potential to overcome the variation in responses observed in delivering live bacteria.

## Conclusion

The field of the gut microbiome and immunotherapy is rapidly expanding with new mechanisms of action, new bacterial species, new bacterial metabolites, and new cancers impacted by the microbiome discovered every year. With the gut microbiome becoming a common topic in news outlets, many patients wonder what they can do to improve their microbiome. At least for melanoma, patients who do not take over-the-counter probiotics and eat more than 20 g of fiber a day have increased overall survival (14). However, we expect this rapidly expanding field to have more actionable answers in the near future. While fecal transplants may provide a stopgap for patients who have failed other therapies, understanding the specific bacterial metabolites that promote antitumor immunity and their mechanisms of action will be important to design safe, predictable, and effective immunotherapies.

## Figures and Tables

**Table 1:**
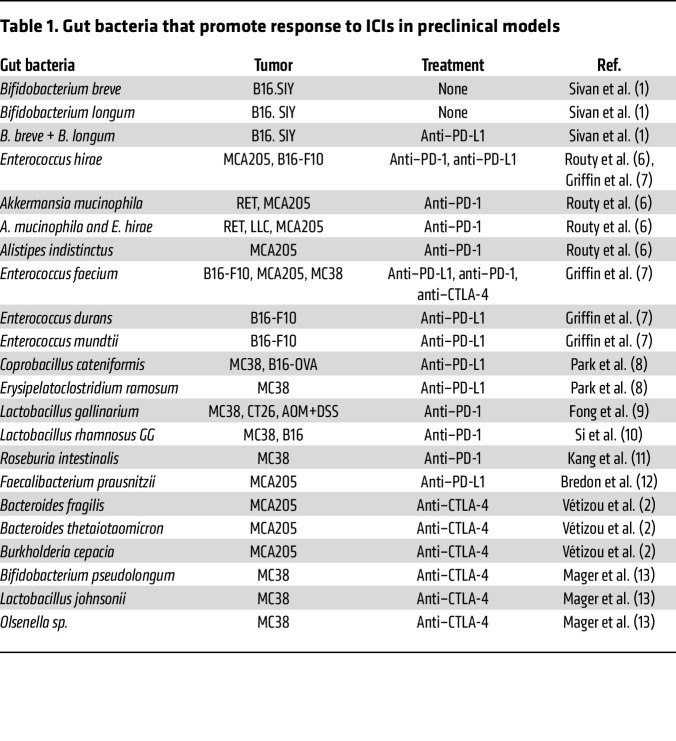
Gut bacteria that promote response to ICIs in preclinical models

## Version 1 02/03/2025

Electronic publication
